# Postpartum septic symphysitis, a rare condition with possible long-term consequences: a cohort study with long-term follow-up

**DOI:** 10.1186/s12884-021-04023-w

**Published:** 2021-11-16

**Authors:** Helen Elden, Monika Fagevik Olsen, Nasrin Farah Hussein, Lisa Wibeck Axelsson, Verena Sengpiel, Michael Ullman

**Affiliations:** 1 Institute of Health and Care Sciences, Sahlgrenska Academy, University of Gothenburg, Box 457, 405 30 Gothenburg, Sweden; 2 Region Västra Götaland, Sahlgrenska University Hospital, Department of Obstetrics and Gynecology, Gothenburg, Sweden; 3 Region Västra Götaland, Sahlgrenska University Hospital, Department of Orthopedics, Gothenburg, Sweden; 4 Department of Health and Rehabilitation/Physiotherapy, Institute of Neuroscience and Physiology, Sahlgrenska Academy, University of Gothenburg, Gothenburg, Sweden; 5 Region Västra Götaland, Sahlgrenska University Hospital, Department of Occupational Therapy and Physical Therapy, Gothenburg, Sweden; 6 Department of Orthopaedics, Institute of Clinical Sciences, Sahlgrenska Academy, Gothenburg University, Gothenburg, Sweden

**Keywords:** Lumbopelvic pain, Osteitis pubis, Osteomyelitis, Pelvic girdle pain, Postpartum septic symphysitis, Postpartum septic arthritis, Symphysitis pubic

## Abstract

**Background:**

Postpartum septic symphysitis (PPSS) is defined as acute onset of severe pain around the symphysis, restricted movement, fever, and elevated inflammatory parameters. It is a rare but serious condition requiring urgent diagnosis and treatment. The aim of this study was to describe the incidence, symptoms, diagnosis, treatment, and long-term follow-up of PPSS.

**Methods:**

This follow-up study included 19 out of 21 women diagnosed with PPSS from 1989 to 2017 at one tertiary care hospital in Sweden. Clinical data were retrieved from hospital records and compared to those retrieved from a regional registry. Women completed a postal questionnaire, and those who reported lumbopelvic pain (LPP) were offered a clinical examination.

**Results:**

1) PPSS was diagnosed after a normal postpartum period of 24 to 50 h by blood tests (*n* = 19/19), ultrasonography (*n* = 9 /19), computer tomography (*n* = 8/19) or magnetic resonance imaging (*n* = 16/19) Treatment included aspiration of symphyseal abscesses, i.v. antibiotics and different physiotherapeutic interventions. Women with PPSS more frequently were primiparous (*n* = 14/19, *p* = 0.001), had an instrumental delivery (*n* = 14/19, *p* = 0.003), longer time of active labour (*p* = 0.01) and second stage of labour (*p* = 0.001) than women in the regional registry. 2) Ten out of 19 (52%) women reported LPP at follow-up. These women more often suffered impaired function related to LPP (Pelvic Girdle Questionnaire, 27 versus 0, *p* < 0.0001), a poorer health-related quality of life (EuroQol-5 dimensions *p* = 0.001 and EuroQol-visual analogue scale, 65 mm versus 84 mm, *p* = 0.022) and higher levels of anxiety and depression (Hospital Anxiety Depression Scale (HADS) HADS-Anxiety, 7 versus 2, *p* = 0.010; and HADS-Depression, 1 versus 0, *p* = 0.028) than women with no pain. 3). Of the eight women who were clinically assessed, one had lumbar pain and seven had pelvic girdle pain (PGP).

**Conclusions:**

In the largest cohort of patients with PPSS to date, primiparas and women with instrumental vaginal delivery were overrepresented, indicating that first and complicated deliveries might be risk factors. Approximately half of the women reported PGP at follow-up, with considerable consequences affecting health-related quality of life and function decades after delivery. Prospective multicentre studies are needed to establish risk factors, long-term consequences, and adequate treatment for this rare pregnancy complication.

## Background

Postpartum septic symphysitis (PPSS) is a rare but incapacitating condition [1–11]. PPSS is defined as pain around the symphysis in combination with signs of infection (i.e., fever, malaise), elevated laboratory tests indicating a bacterial infection and pain on movements including walking. Computer tomography (CT) and/or magnetic resonance imagining (MRI) and/or ultrasonography should demonstrate an inflammatory process in or adjacent to the symphysis. The onset of symptoms is acute after an apparently normal delivery and early postpartum period. The pathophysiology linking PPSS to childbirth is unknown [1]. It has been suggested that trauma to the pelvic soft tissues occurring during vaginal delivery or caesarean section could facilitate colonisation and the contiguous spread of bacteria in predisposed women, but no particular obstetric risk factors have been reported [1].

### Diagnosis

Diagnosis of PPSS is often missed or delayed due to the rarity of the condition and its variable presentation. In the 11 previously published case reports that describe conditions in accordance with PPSS, diagnosis was based on clinical symptoms as well as radiological and laboratory tests that indicated a septic condition [1–11]. Most of these cases were preceded by a normal vaginal delivery. However, in one case, the delivery was complicated by shoulder dystocia [2]. One case of osteomyelitis of the pubic symphysis occurred in gestational week 37 and resulted in an emergency caesarean section; in another case, PPSS debuted at 36 gestational weeks. This woman had experienced anterior Pelvic Girdle Pain (PGP) since week 28 and used a walker from gestational week 32 [4, 7]. Additionally, Cosma et al. [1] reported a case of a 39-year-old woman (3 para) with gestational diabetes mellitus who developed signs of PPSS 12 h postpartum of a macrosomic foetus weighing 4530 g without complications during labour.

### Differential diagnosis

In the literature, several conditions causing pain around the symphysis have been described. Inflammatory conditions such as osteitis pubis [12] and postpartum pubic symphysis diastasis [1, 13] must be distinguished from infectious complications such as PPSS. Some conditions similar to PPSS are osteomyelitis of the pubic symphysis [1, 6], septic arthritis of the symphysis (also called pubic osteomyelitis, osteomyelitis of the pubic bone or pubic symphysis), and rare orthopaedic infections, accounting for less than 1–2% of all haematogenous osteomyelitis. In a review of 100 cases, only two cases of septic arthritis of the pubic symphysis appeared after delivery [11]. Septic arthritis has been described in a fractured pubic bone [14]. The diagnosis of septic arthritis requires a bone scan or radiological signs of engagement of the pubic bone, i.e., oedema or irregular indentations of the adjacent joint surfaces, which are not observed in the very acute phase [15].

Septic symphysitis has been reported to be related to female incontinence surgery, pelvic surgery, pelvic malignancies, intravenous drug use, trauma, cardiac catheterization, and impaired venous circulation in the pubic vein [11, 16–21]. The most common pathogens responsible for septic symphysitis are *S. aureus*, *P. aeruginosa*, and *S. mitis*.

Initially, it might be difficult to distinguish PPSS from PGP, a pregnancy-related condition present in 20% of pregnant women worldwide [22, 23]. PGP is mainly located between the posterior iliac crest and the gluteal fold, particularly in the vicinity of the sacroiliac joints, separately or in conjunction with pain in the symphysis [22]. PGP most often starts during the second trimester of pregnancy but can also begin after delivery and may persist for a long time thereafter [24]. Other differential diagnoses include neurological damage caused by delivery, resulting in pain and disability during the postpartum period; diastasis symphysis pubis, a non-infectious separation of the symphysic joint [16]; pelvic haematoma or abscess; genitourinary injuries; and insufficiency fractures [25]. Another condition is puerperal endometritis, characterized by pelvic pain, uterine or parametrial tenderness, maternal tachycardia, foul smelling lochia and elevated leucocyte count (≥109 g/L) [26].

### Treatment

Urgent diagnosis of PPSS is crucial to initiate appropriate treatment. An abscess causing clinical deterioration under antibiotic treatment necessitates aspiration and drainage [11]. After the abscess is aspirated and/or blood culture samples are collected, treatment with broad-spectrum antibiotics covering group *G streptococci*, *staphylococci* and *diptheroids* should be started. Analgesics including paracetamol and nonsteroidal anti-inflammatory drugs (NSAID) as well as bed rest should be prescribed [1, 11, 20, 21].

### Prognosis

The immediate outcome of PPSS is reported to be excellent in most cases if prompt treatment is established [1], but there are no long-term follow-up studies of women with PPSS and no consensus or guidelines for the management of these patients during and after the acute phase. Thus, the aim of this study was to describe the symptoms, diagnosis, treatments, possible risk factors and long-term follow-up for all women diagnosed with PPSS in a single tertiary care hospital in Sweden over a period of 28 years.

## Methods

### Setting/study design

This cohort study with long term follow-up is based on women diagnosed with PPSS at Sahlgrenska University Hospital (SU), Gothenburg, Sweden. The Hospital has approximately 10,000 deliveries/year. The study comprises three different parts: a retrospective hospital record-based analysis, a postal questionnaire and a clinical examination offered to women reporting LPP in the questionnaire.

### Participants

Eligible patients were all 21 women diagnosed with a septic condition in the symphysis, with no other cause than the recent delivery at SU 2000–2018. The name PPSS has been suggested during the study period, as a condition that excludes the other above-mentioned differential diagnoses.

### Initial diagnosis

Diagnostic criteria for PPSS at the time of the study were acutely occurring pain around the symphysis in combination with signs of infection (i.e., fever, malaise), elevated laboratory tests indicating a bacterial infection and pain on movements including walking. Clinical routine during the study period was that women with infectious complications in the pelvis after delivery were referred to the department of orthopaedics, at Sahlgrenska University hospital where basically only one single orthopaedic surgeon was consulted regarding diagnosis and treatment of the women with PPSS during the study period.

**Fig. 1:**
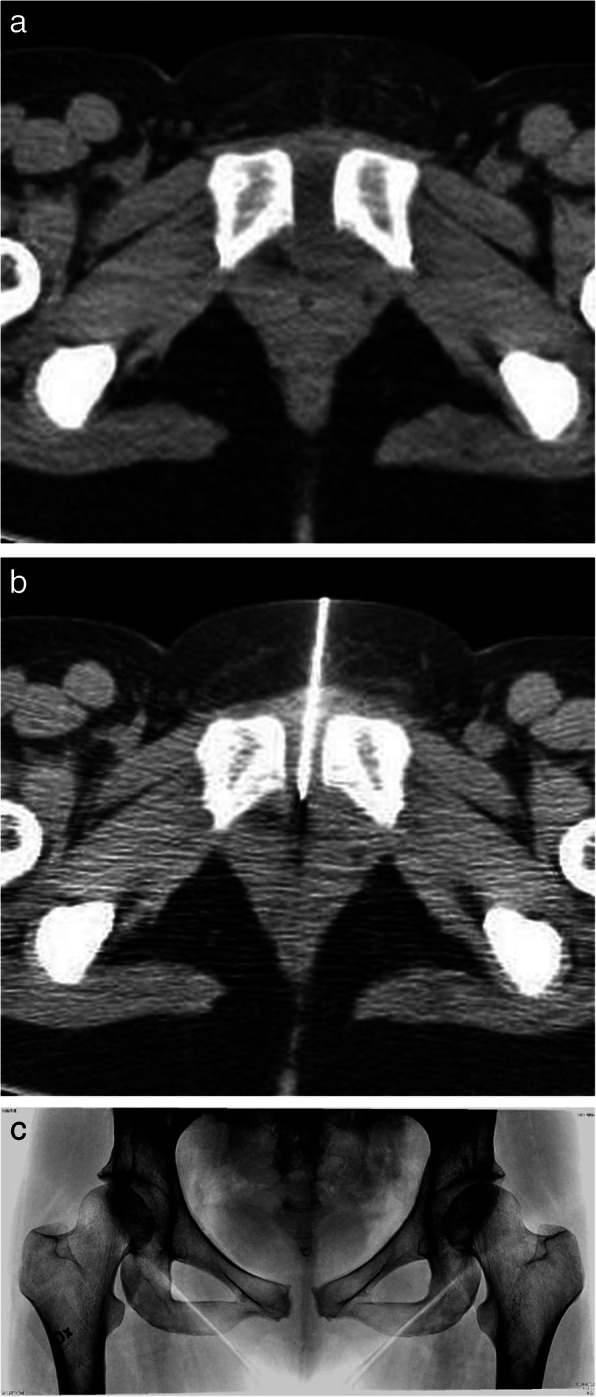
**a** A 38-year-old year woman. First and normal delivery of a foetus weighing 4530 g. Post delivery onset of pains around the symphysis, malaise and inability to move and walk. High fever and CRP 270. CT scan showed an abscess of the symphysis 4 × 2 × 5 cm. *CRP*, c-reactive protein, *CT*, Computer Tomography. **b** The symphysis was aspirated under ultra-sound guidance acutely. A new aspiration of 6 ml pus, was done under CT scan guidance five days later. Both cultures revealed Staph. Aureus. Treatment was initiated from day two. *CT*, Computer Tomography. **c** Six months post-partum, the patient had still symphyseal pains on walking. Radiogram shows a widened symphysis

**Fig. 2:**
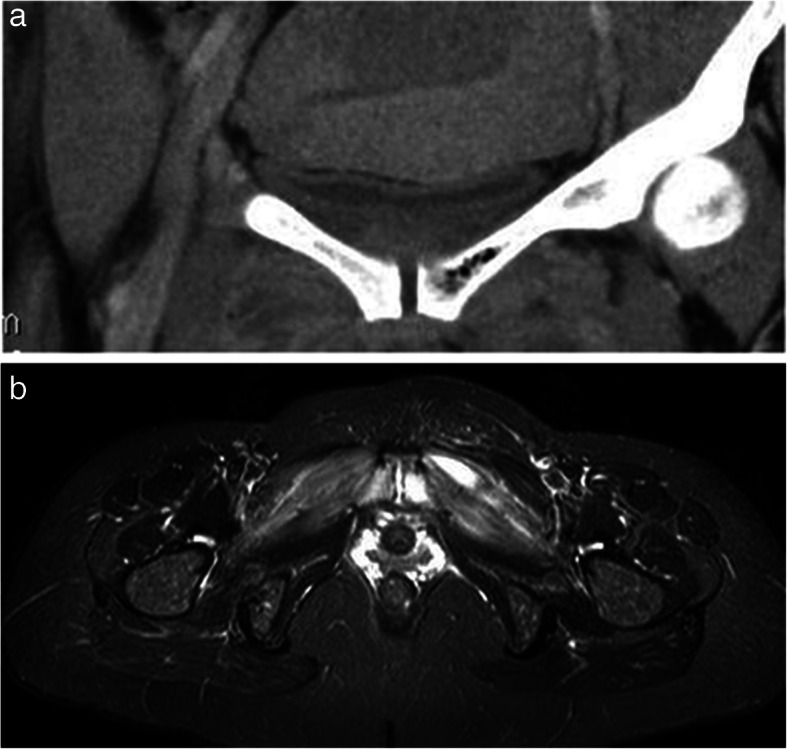
**a** A 35-year-old woman, normal first delivery of a foetus weighing 4530 g. Increasing pain around the symphysis firsts day postpartum. Readmitted nine days postpartum with pain around the symphysis, and inability to walk. Temp: 38.5°, CRP 180. CT scan shows infected haematomas in and around the symphysis and gas in the left superior ramus, indicating septic osteomyelitis, and also bilateral abscesses in the adductor muscles. Broad-spectrum antibiotics and NSAIDs were given, but symptoms receded. CRP slowly. *CRP*, C-reactive protein, *CT*, Computer tomography, *NSAIDS*, non-steroid anti-inflammatory drug. **b** Two weeks later MRI shows receding abscesses in the left abductor muscles of the pelvis. *MRI*, magnetic resonance imagining

**Fig. 3:**
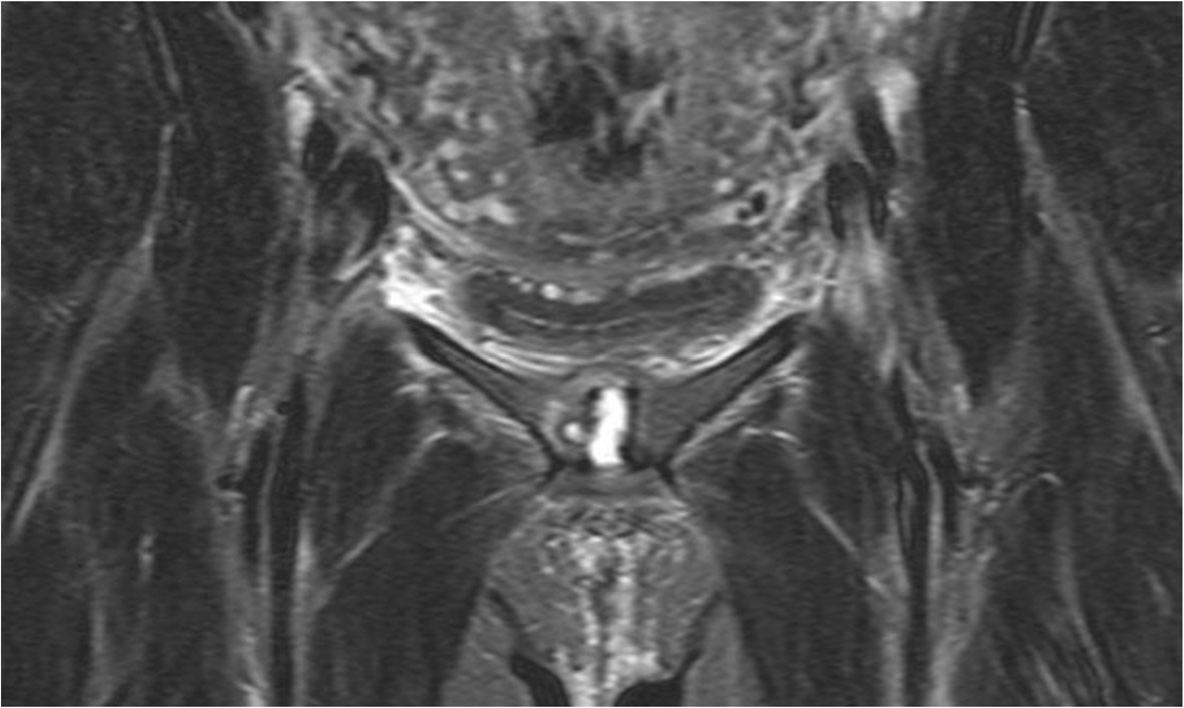
Coronal plane MRI showing oedema in the distracted symphysis with a cavity in the right periarticular subchondral bone, indicating a spread of infection from the cartilaginous disc

**Fig. 4:**
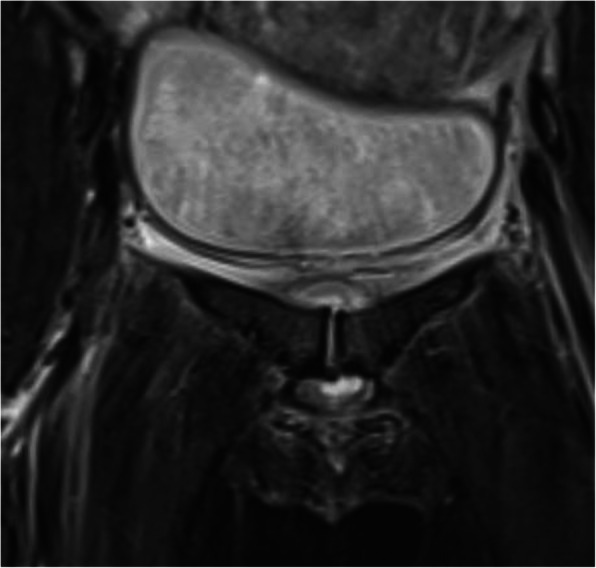
Oedema around the symphysis, spreading cranially, dorsally and caudally

**Acute clinical findings** The obstetricians in charge were recommended to investigate the symphysis with ultrasound, and to refer the patient for a CT, as in (Fig. 1a) and/or MRI scan, and if an oedema or abscess was found, initiate an aspiration guided by ultrasound or CT (Fig. 1b). The abscess can spread into the periarticular subchondral bone of the ramus (Fig 2). The abscess can develop to an osteomyelitis of the pelvis (Figs. 2a and 3), or an intramuscular abscess in the hip adductor muscles (Fig. 4b). One case developed a septic arthritis of the right hip joint, with a probable origin in the septic symphysitis. The pus had to be surgically evacuated from the joint, and a secondary osteoarthritis developed.

Twenty of the 21 women diagnosed with PPSS, had a valid address, and received a postal invitation to participate in the study. The letter, mailed in March 2018, contained information about the study, a previously used questionnaire about long-term follow-up in women with PGP [24] and a written informed consent form. The questionnaire was based on a reliable and validated instrument to assess lumbo-pelvic pain (LPP) that was used in an earlier longitudinal follow-up study of women with PGP during pregnancy [24]. Two reminders were sent.

### Hospital records

The hospital records of women who consented to participate were analysed to determine the baseline characteristics of parity, age at delivery, first recorded body mass index (BMI) in pregnancy and delivery outcomes (one hospital record was missing). Available pooled obstetric data of all women in the same region during the same period were retrieved from the Swedish National Board on Health and Welfare and used for comparison [27].

### Follow-up questionnaire

The presence of self-reported LPP at follow-up was assessed by one question derived from a modified multi-centre Delphi study [28]: “Have you experienced lumbar pelvic pain with or without radiation into one or both legs during the past 4 weeks?” The pain should have been bad enough to limit usual activities or cause changes in daily routines for more than 1 day. The variables of age, parity and education level were also reported on the questionnaire, as well as patient-reported outcomes, such as physical activity, and measurements of function were obtained with the Pelvic girdle questionnaire (PGQ) [29]. The PGQ [29, 30] is a self-administered questionnaire consisting of 25 items; 20 items evaluate consequences from PGP on an activity subscale, and the symptoms are assessed on a 5-item symptom subscale. The scores were subsequently converted to percentages ranging from 0 (no disability) to 100 (severe disability). Health-related quality of life was measured with the European quality of life measure (the EuroQol- 5 dimensions (EQ-5D) and the EQ-visual analogue scale (EQ-VAS) [31, 32]. The EQ-5D assesses five dimensions of HRQL: mobility, self-care, activities of daily life, and pain. Levels of anxiety and depression were measured. For each dimension, the women selected one of three possible levels (none, mild to moderate and severe). This descriptive system contains 243 combinations or index values to assess the state of health. The total score range is from − 0.43 to 1.0, in which − 0.43 is the lowest and 1 is the highest health state. For a normal population, the average value is 0.8–0.9 [32]. The EQ-5D VAS is a vertical VAS (0–100 in which 0 is the lowest conceivable health state, and 100 is the optimal health state) [33]. Levels of anxiety and depression were measured with the Hospital anxiety and depression scale (HADS) [34], and self-efficacy was measured with the General self efficacy scale (GES) [35]. The HADS is a 14-item scale that evaluates anxiety and depression in people with physical health problems. Seven items relate to anxiety (HADS-A), and 7 items relate to depression (HADS-D). Each item on the questionnaire is scored from 0 to 3, for totals scores of 0 to 21 for both anxiety and depression. A cut-off score of 8/21 for both anxiety and depression has been identified [36]. For anxiety, this tool has a specificity of 0.78 and a sensitivity of 0.9. For depression, this tool has a specificity of 0.79 and a sensitivity of 0.83 [36]. Self-efficacy theory refers to one’s ability and belief in one’s ability to cope with stressful situations. According to Bandura [37], self-efficacy comes from past experiences with specific situations, experiences learned from others, social persuasion, and physiological and affective states. Pain catastrophising was measured with the Pain catastrophizing scale (PCS) [38]. It is a self-reported measurement tool consisting of 13 items scored from 0 to 4, resulting in a total possible score of 52 [38]. The three subscales of magnifications, rumination, and helplessness reveal different dimensions of the same underlying content. Catastrophizing has been defined as an irrational forecast of future events [39], and pain catastrophizing refers to an individual’s negatively exaggerated cognition of a painful situation; it has been measured during childbirth and postpartum recovery [40]. Women reporting LPP were also asked questions about the use of analgesics or sick leave due to this type of pain.

### Clinical examination

All women reporting LPP were offered an appointment with a physiotherapist specialised in PGP. Examination of the pelvic joints was performed according to defined guidelines [22]. Tests for range of motion in the back and hip, skin sensation in the affected area and strength of the hip muscles were examined. These patients were screened for hypermobility with a specific questionnaire [41].

### Statistical analysis

The clinical, record-based data of all women with PPSS were compared with data in the available literature and the incidence in the general population of women who gave birth in the same Swedish health care region during the same period [27]. Clinical, record-based data were then compared between the groups of women with and without reported LPP at follow-up. Patient-reported outcomes are presented separately and were compared between the two groups with and without reported LPP to study the impact of LPP on different aspects of health and well-being at follow-up. Continuous variables are presented as median, minimum, and maximum values, and categorical variables are presented as numbers and percentages. As the data were not normally distributed and the study population was small, non-parametric statistics were used. For comparison between groups, Fisher’s exact test was used for dichotomous variables (if *n* > 0/group), Mantel-Haenszel’s chi-square exact test was used for ordered categorical variables, the chi-square test was used for non-ordered categorical variables, and the Mann-Whitney U-test was used for continuous variables. All significance tests were two-sided and conducted at the 5% significance level. All statistical analyses were performed with SPSS, version 24.

## Results

**Fig. 5:**
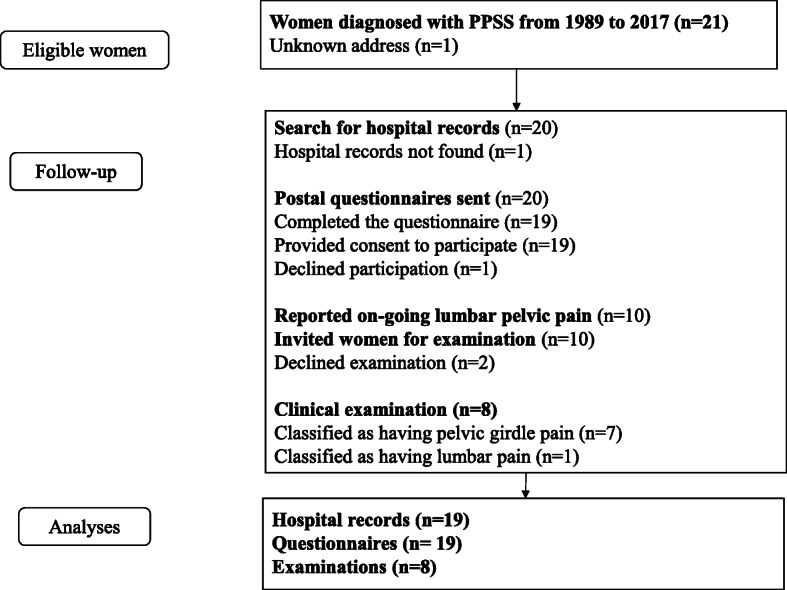
The flow-shart of the study

There were 21 cases of PPSS identified during the 28-year period (range 1 to 28 years (mean 8 years) with approximately 10,000 deliveries per year in the Gothenburg region. This indicates a PPSS incidence of 0.01%. Nine-teen of 20 women returned their written consent forms and completed the questionnaire. Hospital records of 19 women who consented to participate were retrieved to determine the baseline characteristics, whereas records were unavailable for one woman. Ten women reported LPP at follow-up and were offered a physical examination including an established pain provocation assessment [22]. Two women declined the visit; thus, eight women were examined. All eight women reported chronic LPP, i.e., that LPP had always been present since the postpartum period of the index pregnancy. Figure 5 shows the progress of patients throughout the study.

### Participant characteristics

Table 1 shows the baseline characteristics obtained from the hospital records of the women with PPSS; the women were grouped according to the presence or absence of LPP at follow-up and compared to those in the same health care region during the same period, i.e., 1989 to 2017 as reported by the Swedish National Board on Health and Welfare. The average age for women with PPSS at delivery was 33 years, and that of the general population during the same period was 29.6 years. However, there were more primiparous women (*p* = < 0.001) and more women with a vaginal instrumental delivery (*p* = < 0.003) in the PPSS group than in the women who gave birth in the same area during the same period (Table 1). Additionally, more women with PPSS reported PGP during the index pregnancy (*n* = 9/19) and had classified PGP at follow-up (*n* = 8/19) than stated in the literature [22, 24, 42]. One of the women required an emergency caesarean section.

**Table 1:** Characteristics for women diagnosed with PPSS and all pregnant women in VGR^a^

Variable	All women with PPSS (*n* = 19)	All pregnant women in VGR (min-max)^a^	Pain (*n* = 10)	No pain (*n* = 9)	*P*-value for comparison of persistent pain versus no pain at follow-up	*P*-value for comparison of all pregnant women in VGR^a^ versus all women with PPSS during pregnancy
Primipara during index delivery	14 (74)	(43.5) (39.7 in 1994 to 46.3 in 2008)	8	6	0.444	< 0.001
2nd/3rd delivery	5 (26)		2	3		
Pelvic girdle pain during index pregnancy*	9 (47)	–	4	5	1.000	
Age at delivery, years	33 (27–43)	29.6 (28.0 in 1989 30.5 in 2017)	33 (28–34)	32 (27–43)	0.768	
Body mass index at first antenatal care visit	25.4 (17.0–34.9)	24.4 (23.4 in 1992 to 25.1 in 2017)^b^	26.1 (20.3–29.3)	24.8 (17.0–34.9)	0.624	
Gestational weeks at delivery, min-max	40 (34–42)	–	40 (38–42)	40 (34–4)	0.666	
Spontaneous contractions	15 (79)	(89.3) (82.7 in 2017 to 92.3 in 1991)^c^	7	8	0.582	
Induction of labour	4 (21)	10.6 (7.7 in 1991 to 17.3 in 2017)^c^	3	1	0.582	0.408
Use of oxytocin	9		4	5	1.000	
Missing data	2		2			
Established contractions to delivery, hours, (min-max)	8.4 (1.5–18)		10.6 (3.5–18)	6.4 (1.5–11.8)	0.152	
Missing data	5		3	2		
Duration of second stage of labour, minutes, (min-max)	38 (7–310)		36 (16–60)	38 (7–310)	0.864	
Missing data	4		4	–		
Birth position						
On the side	1		1	–	0.281	
Lithotomy	8		3	5		
Semi-sitting	3		–	3		
Dorsal Recumbent	5		4	1		
Missing data	2		1	1		
Occiput anterior position at delivery	17		8	9	1.000	
Missing data	2		1	1		
Vaginal instrumental delivery	4	6.4 (5.0 in 1989 and 2017 to 7.7 in 2009)	2	2	0.509	0.003
Mid-vacuum extraction	3		2	1	NA	
Low-vacuum extraction	1		–	1	NA	
Caesarean section (emergency)	1	13.9 (10.0 in 1991 to 16.7 in 2016)	1	–		
Total bleeding, ml	375 (200–1400)		350 (200–1100)	425 (300–1400)	0.508	
Episiotomy	3		1	2	0.582	
No vaginal tear	4		1	3	0.213	
Vaginal tear	10 (53)		4	6	0.637	
Perineal tear	8 (4)		1	7	0.620	
Third- or fourth-degree tears	2 (1)		1	1	1.000	
Characteristics of newborns						
Weight (g), min-max	3800 2335–4610	3516.7 (3488.7 in 2008 in to 3541.8 in 1994)	3980 2980–4610	3670 2335–4610	0.4888	
Sex (girl)	6		3	3	1.000	
Sex (boy)	13		7	6	1.000	
Apgar score ≤ 7 at 5 min	2	(1.2) (0.9 in 1994 to 1.41996)	2	–	0.259	NA
Perinatal mortality	1	0.79 (0.60 in 2015 to 1.05 in 1990)	1	–	1.000	NA

Characteristics for women diagnosed with PPSS and all pregnant women in the same health region during the same period. Data from hospital records. *PPSS*, post-partum septic symphysitis, *VGR*, Västra Götaland Region. *SoS data*^a^, Data from the Swedish National Board on Health and Welfare *Patient-reported data. Median (min-max) or n (%, only for the whole group). Fisher’s exact test or Chi-square test were used for categorical variables and the Mann-Whitney U-test was used for continuous variables

*NA* not applicable

^a^SoS data VGR 1989–2017: https://sdb.socialstyrelsen.se/if_mfr_004/val.aspx

^b^Data available from 1992 onwards

^c^Data available from 1991onwards and for vaginal deliveries only

### Clinical presentation at diagnosis (hospital record-based data)

Table 2 shows descriptive data on symptoms, diagnosis and treatment from hospital records for all women and separated for women with and without LPP at follow-up.

**Table 2:** Hospital records: Descriptive data on symptoms, diagnosis and treatment of PPSS

Variable	All women (*n* = 19)	Pain (n = 10)	No pain (*n* = 9)
Year of diagnosis			
1989–1999	3 (16)	3 (30)	–
2000–2010	7 (37)	2 (20)	5 (56)
2011–2018	9 (47)	5 (50)	4 (44)
Age, years	33 (27–43)	33 (28–43)	33 (27–43)
Last recorded BMI in pregnancy, kg/m^2^	27 (20–36)	29 (23–36)	25 (20–34)
Symptoms	38.0 (64.8)	24.6 (29.0)	49.8 (81.9)
PPSS debut, hours after delivery, min-max	0–264.0	8.2–72.0	0–264.0
Pain location			
Symphysis pubic	14 (74)	7	7
Sacroiliac joint/s plus hip joints	11 (58)	5	6
Temperature ≥ 37.5 °C	15 (79)	7 (70)	8 (89)
≥ 37.5 °C, days	3.3 (1–7)	3.2 (1–7)	3.5 (1–6)
Diagnosis			
Ultrasound	9 (47)	3	6
CT	8 (42)	4	4
MRI	16 (84)	7	9
Blood tests	19 (100)	10	9
CRP > 5 at diagnosis	17 (89)	8	9
Leucocytes at diagnosis ×10^9^/L	15.4 (6.3–20)	10.8 (7.3–20)	15.8 (6.3–20)
Blood culture	10 (53)	6	4
Wound exudate cultivation	2	1	1
Aspiration of abscess	1	1	0
Positive blood culture or wound exudate cultivation	3	2	1
Treatment			
Antibiotics	19 (100)	10	9
Different Physiotherapeutic interventions	14 (74)	6	8
Wheel-chair, crutches, walker	13 (68)	6	7
Pelvic belt	7	4	3
Pain killers, paracetamol	16 (84)	7	9
Pain killers, NSAIDs	11 (58)	4	7
Pain killers, opioids	13 (68)	6	7
Patient-controlled analgesia	1	1	0
TENS	2	0	2
Follow-up visit to physician	14 (74)	7	7

Data from hospital records. BMI body mass index *PPSS* post-partum peptic symphysitis; *MRI* magnetic resonance imaging, *CT* computed tomography, *CRP* c-reactive protein, *NSAID* non-steroidal anti-inflammatory drugs, *TENS* transcutaneous nerve stimulation. Median (min-max) or n (%, only for the whole group). Fisher’s exact test or Chi-square test were used for categorical variables and the Mann-Whitney U-test was used for continuous variables. All *P*-values are ≤0.05

**Fig. 6:**
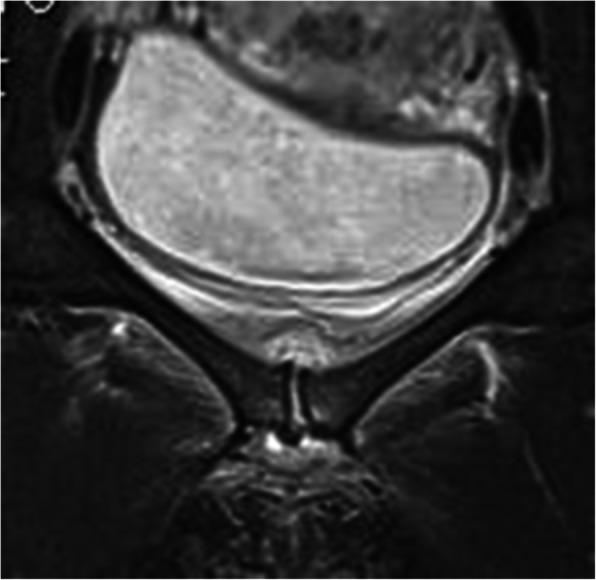
MRI showing oedema of the symphysis protruding both cranio-dorsally, approaching the urinary bladder, as well as caudally close to the urethra

PPSS was diagnosed after delivery and an uncomplicated post-puerperal period of 24 to 50 h. Diagnosis was established by blood tests (*n* = 19), magnetic resonance imagining (MRI) (*n* = 15/19), ultrasonography (*n* = 9/19), or computer tomography (CT) (*n* = 8/19) (Figs. 1, 2, 3 and 6).

Blood culture or wound exudate swabs were positive in three women, presumably because i.v. antibiotics were administered before culture samples were collected. Treatments included i.v. antibiotics, aspiration of symphyseal abscesses (*n* = 2/19) and physiotherapy.

### Comparison between women with and without LPP at follow-up (hospital record-based data)

Comparisons of data from hospital records of women who reported LPP and women without LPP at follow-up indicated that there were no differences in baseline characteristics or the diagnosis of PPSS (Tables 1 and 2).

The mean time from delivery to the onset of symptoms was 25 h in the women with LPP at the time of the follow-up and 50 h in the women with no LPP. Of the women with no LPP, three women had given birth once, two women had given birth twice, and one woman had given birth three times after PPSS. Of the women with LPP, six women had given birth once, and one woman had given birth twice after PPSS. There were no differences in time since PPSS onset, education level, or physical activity level between women with LPP and women without LPP (Table 3).

**Table 3:** Characteristics and patient-reported outcomes at follow-up

Variable	All women (*n* = 19)	LPP (*n* = 10)	No LPP (*n* = 9)	*P*-value
Time since PPSS, years, min-max	8 (0.5–22)	8 (0.5–22)	8 (2–12)	0.456
Highest education level				
Primary/lower secondary school	–	–	–	
Upper secondary school	6 (32)	4	2	
Post-secondary vocational education and training	2 (11)	1	1	
University degree	10 (53)	5	5	0.655
Other	1 (5)	0	1	
Physically active ≥30 min, days/week	8 (0–7)	5 (1–7)	3 (0–7)	0.395
Sick-leave due to persistent PPSS	2 (11)	2	0	0.474
Sick-leave due to other	1 (5)	0	1	
PGQ	9 (0–72)	27 (9–72)	0 (0–6)	< 0.001
EQ-5D score	0.880 (0.578–0.969)	0.740 (0.578–0.8780)	0.914 (0.868–0.969)	< 0.001
EQ–VAS	80 (30–100)	65 (30–85)	84 (66–100)	0.022
HADS-A, sum of scores	5 (0–9)	7 (0–17)	2 (0–9)	0.010
HADS-A > 8	5	4	1	
HADS-D, sum of scores	3 (0–9)	4.5 (1–9)	1 (0;-)	0.028
HADS-D, > 8	1 (5.26)	1	0	
PCS	9 (3–40)	8.5 (3–35)	9 (1–40)	0.968
GSE (half-scale)	30.5 (23–39)	29.5 (23–37)	32 (28–39)	0.203

Questionnaire data. *LPP* lumbar pelvic pain, *PPSS* post-partum septic symphysitis, *PGQ* pelvic girdle questionnaire, *EQ-5D* score euroqol 5-dimension score, *EQ–VAS* euroqol visual analogue scale, *HADS-A* hospital anxiety depression scale-anxiety, *HADS-D* hospital anxiety depression scale-depression, *PCS* pain catastrophizing scale, *GSE* general self-efficacy scale. Median (min-max) or n (%), only for the whole group

Fisher’s exact test or Chi-square test or Mantel-Haenszel’s chi-square were used for categorical variables and the Mann-Whitney U-test was used for continuous variables

### Patient-reported outcomes (postal questionnaire data)

Nineteen women completed the follow-up questionnaire. Women with LPP stated that LPP impaired function (PGQ, *p* = < 0.0001) and caused a poorer health-related quality of life (EQ-5D, *p* = 0.001, EQ-VAS, *p* = 0.022) and higher levels of anxiety (HADS-A, *p* = 0.010) and depression (HADS-D, *p* = 0.028) than women with no LPP (Table 3). Moreover, four of 10 women with LPP reported PGP during the index pregnancy in the questionnaire (Table 1). These women also described in the open-text answer that PPSS affected their daily life during the acute phase as well as a prolonged period after delivery. Stated problems were inability to care for their new-born baby, play with the infant, participate in everyday life activities such as shopping, cleaning, washing, and gardening and participate in physical exercise and sports.

### Clinical examination at follow-up

**Fig. 7:**
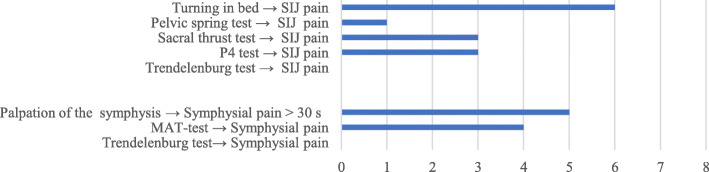
Positive Pain Provocation Tests in women with LPP at follow-up. Palpation: Pain > 30 s after provocation of the symphysis. *P4*,Posterior pelvic pain provocation test. *SI joints* sacroiliac joints, *MAT-test simulates pulling a mat*

Figure 7 shows the number of positive pain provocation tests and Table 4 presents the results from pain on palpation and the classification of LPP according to the clinical examinations. Of the eight women who were clinically assessed, one was classified as having lumbar pain, and seven were classified as having PGP; six of these seven women had painful symphysis. Three women had considerably decreased joint mobility, with four cases in the hips and one case in the lumbar spine, and four women fulfilled the criteria for hypermobility [41]. All women indicated pain when the structures in and around the pelvic girdle were palpated. One woman reported decreased sensitivity in the skin around the symphysis, and two women reported pelvic floor muscle dysfunction. All eight women who were examined stated that their pain had been present since the postpartum period of the index pregnancy, and none of these women had PPSS twice. Three women with classified PGP stated that their thighs had been forcefully abducted during delivery by an obstetrician or a midwife.

**Table 4:** Clinical findings in women with lumbopelvic pain at follow-up

Variable	*n* = 8
Pain on palpation	
Gluteal muscles	4
Piriformis muscles	6
Tendons in the groins	5
Tendons to the adductor muscles	4
Trochanter major	8
Classification	
Only painful symphysiolysis	3
One-sided sacroiliac pain	1
One-sided sacroiliac pain + symphysial pain	2
Double-sided sacroiliac pain	–
Pelvic girdle syndrome	1
Other pain (back pain)	1

Values are n. *Pelvic girdle syndrome*, symphysial pain+ double sided sacroiliac pain

## Discussion

In this study of the largest cohort of PPSS to date, the PPSS-incidence was 0.01%.

Primiparity and vaginal instrumental delivery were more common in women with PPSS than in the general population. Fewer women in the PPSS group with continuous pain had given birth after the initial PPSS event than women with no pain. This supports our findings that primiparity is probably an important risk factor for PPSS. Additionally, PGP during the index pregnancy (*n* = 9/19) was more common in the PPSS group than in women in the literature [22, 24, 42], indicating that PGP may pre-dispose women to PPSS. This is also supported by case studies reported by Lawford et al. [7] and Gamble et al. [4] of women with pubic pain and PPSS, with PPSS debut in gestational weeks 36 to 37.

### Clinical outcomes in relation to earlier studies

The symptoms, diagnosis and treatment of PPSS were in accordance with those in published case studies [1, 10, 11, 20, 21]. However, our result differs from those of Cosma et al.’s [1] review, which reported that the outcome was excellent in most cases when prompt treatment was established. This may be due to differences in the characteristics of the deliveries in Cosma et al.’s [1] review compared to our cohort study, which indicate that first and complicated deliveries might be risk factors. In Cosma et al.’s [1] study, only one woman had a complicated vaginal delivery, i.e., shoulder dystocia, which required episiotomy, suprapubic pressure and McRobert’s manoeuvre. Furthermore, data on other delivery outcomes, such as parity, length of delivery, birth position, etc. were not reported, and no follow-up was performed [1]. In our cohort, however, four women had vaginal instrumental deliveries, three women had episiotomies, and two women had third- or fourth-degree tears. These factors may have contributed to the spread of different infectious agents resulting in acute infection of the symphysis.

### Long-term follow-up in women with no LPP

Ten of the 19 women with PPSS had no complaints at follow-up. The results of these women concerning health-related quality of life, function, general self-efficacy and pain catastrophizing were comparable to those reported in the general population [43–46].

### Long-term follow-up in women with remaining LPP compared to women without LPP

Approximately 50% of the women reported persistent LPP at follow-up, with a considerably decreased health-related quality of life compared to the women with no LPP. The instruments to evaluate symptoms and function [29], anxiety and depression [34], health-related quality of life [31] and pain catastrophizing [38] have been used in follow-up studies of women with PGP [24], and the results in women with LPP are in agreement with those reported in women with PGP during and after pregnancy [24, 42]. However, the anxiety and depression scores were only moderately increased compared to those in earlier follow-up studies of women with PGP during pregnancy [24]. The general self-efficacy scores in women with persistent LPP were similar to those reported in the previously mentioned follow-up study on PGP [24] and in the general populations in Denmark and Finland [47]. Additionally, no differences were found in the levels of general self-efficacy and pain catastrophizing between women with and without persistent LPP. Thus, we could not confirm earlier findings suggesting that pain catastrophizing was associated with disability due to chronic back pain [48]. The pain catastrophizing scores in the women in the current study were also similar to those in pain-free individuals in a Dutch study [49] and lower than those in earlier follow-up studies of women with LPP 6 months to 11 years after delivery [24, 42]. Thus, the present study did not indicate that pain catastrophizing is associated with LPP after pregnancy.

### Follow-up visit

Clinical examinations at follow-up visits revealed that LPP was localized in the symphysis pubis and, in some cases, the sacroiliac and hip joints; thus, these cases resembled the non-septic condition of PGP [22]. One woman had lumbar back pain, which is common in the general population [50]. All eight women reported persistent PGP since the index pregnancy at follow-up, and five of them had at least one positive pain provocation test result at the symphysis, which validated the women’s claims.

### Strength and limitations

To the best of our knowledge, this is the largest and most comprehensive study on women with PPSS and the first study to present long-term follow-up data. The strengths of this study are that data from the Swedish National Board on Health and Welfare [27] enabled us to compare characteristics between PPSS patients and the general population and the high response rate (95%), with 19 of 21 eligible women participating. The high response rate may reflect the fact that many of these women received insufficient recognition from healthcare providers when seeking treatment during the post-delivery period. Moreover, the same orthopaedic surgeon (MU) was consulted in all cases.

Another strength is that the cohort consists of all 21 women who were diagnosed with a septic condition in the symphysis, with no other explanation than the recent delivery at Sahlgrenska University Hospital 2000–2018. Moreover, all women with possible pregnancy related complications occurring within 3 months postpartum are followed up at the department of obstetrics. This indicates that few serious infections are missed during the study period. In addition, a strength is that all the women who reported persistent pain were examined by a single physiotherapist specialized in PGP (MFO).

The variables included in the questionnaire are included in the European Guidelines of Diagnosis and Treatment for PGP [22], and the PGQ used is the only condition-specific, reliability tested and validated questionnaire for PGP. It reflects both impaired body function and activities in daily life as well as PGP. Other strengths are that the patient-reported outcomes showed good internal consistency, test-retest reliability, and construct validity when applied in a sample of participants with postpartum PGP [24].

Limitations of this study are the retrospective design and the small number of cases due to the rarity of the condition (21 cases in almost 30 years), which diminishes the possibility of analyzing risk factors for PPSS and predictors of long-term outcomes. An incidence of approximately 1:10000 deliveries require a prospective multicentre approach to increase knowledge about risk factors, causality and outcomes of women with PPSS.

Several women in our study reported that they received insufficient care from healthcare professionals when seeking treatment and that PPSS affected their daily life due to an inability to take care of their newborn baby and participate in everyday life activities during the early postpartum period, as well as later on in their lives. This also has been reported by women with PGP after pregnancy [51]. Thus, it is important to recognize the early symptoms of PPSS. A multidisciplinary team should care for these women, and they should be offered follow-up appointments during convalescence.

On the basis of these results, we suggest the following:

- Newly delivered mothers with unexpected onset of pain in the symphysis region and with acute signs of infection should urgently be referred to a multidisciplinary team including an obstetrician, infection specialist, orthopaedic surgeon and intervention radiologist for immediate diagnosis and treatment of a potential septic infection.
- Prolonged labour, instrumental delivery and/or forced abduction of the thighs and/or the iliac crest might be risk-factors for PPSS.
- Diagnosis should be established by ultrasound, MRI and/or CT scanning of the symphysis; CRP level and leucocyte count; bacterial culture from blood; and aspiration in cases of abscess.
- Acute treatment involves aspiration and/or drainage of abscesses, followed by intravenous broad-spectrum antibiotics until symptoms recede and bacterial cultures are analysed. Peroral antibiotics should be given for at least another 3 weeks.
- During the convalescent period, a physiotherapist with specialised competence in the analysis of PGP should be consulted, and when needed, a pain specialist and/or a psychologist should be consulted for adequate diagnosis and treatment of post-infection pain.

## Conclusions

Primiparity and instrumental delivery were more common in women with PPSS than in the general population, indicating that first and complicated deliveries might be risk factors. Symptoms of septic symphysitis did not appear earlier than 24 h or later than 50 h after delivery. Approximately 50% of the women had persistent LPP, and all women reported that LPP had been present since the postpartum period of the index pregnancy and had considerable consequences on health-related quality of life and function decades after delivery. However, due to the rarity of the condition, uncertainty remains regarding the aetiology of the condition as well as risk factors for PPSS and long-term PGP. Due to an incidence of approximately 1:10000 deliveries, a prospective multicentre approach is required to increase knowledge about the risk factors for and causes of PPSS.

## Data Availability

The datasets generated and/or analysed during the current study are available from the corresponding author on reasonable request.

## Abbreviations

BMI: Body mass index
CT: Computed tomography
CRP: C-reactive protein
EQ-5D: Euroqol-5 dimensions scale
EQ-VAS: Euroqol-visual analogue scale
GES: General efficacy scale
HADS: Hospital anxiety depression score
IV: Intra-venous
LPP: Lumbar pelvic pain
MRI: Magnetic resonance imagining
NA: Not applicable
NSAID: Non-steroid anti-inflammatory drug
PCS: Pain catastrophizing scale
PGP: Pelvic girdle pain
PGQ: Pelvic girdle questionnaire
PGS: Pelvic girdle syndrome
PPSS: Postpartum septic symphysitis
SI-joints: Sacroiliac joints
4p-test: Posterior pain provocation test
